# Schistosomiasis in a Scottish school group after freshwater swimming in Uganda: the need to raise awareness

**DOI:** 10.1099/jmmcr.0.005166

**Published:** 2018-09-25

**Authors:** Sandra L. Currie, Lucy Denvir, Busi Mooka, Kali Perrow, Claire L. Alexander

**Affiliations:** ^1^​ Department of Medical Microbiology, NHS Tayside, Dundee, UK; ^2^​ Directorate of Public Health, NHS Tayside, Dundee, UK; ^3^​ Department of Infectious Diseases, NHS Tayside, Dundee, UK; ^4^​ Travel and International Health Team, Health Protection Scotland, UK; ^5^​ Scottish Parasite Diagnostic and Reference Laboratory (SPDRL), NHS Greater Glasgow and Clyde, UK

**Keywords:** Schistosomiasis, asymptomatic, swimmers’ itch, Katayama fever, praziquantel

## Abstract

**Introduction:**

Schistosomiasis, a travel-related trematode infection, can cause a range of symptoms with potentially life-threatening complications. In this report, we describe an outbreak of schistosomiasis in a Scottish school group that had travelled to Uganda. We discuss the requirement for robust and accurate pre-travel advice, and the importance of raising awareness in travellers, particularly due to the asymptomatic nature of the disease. In addition, we highlight the need to submit a serum sample for laboratory testing on return from endemic regions where freshwater exposure has occurred.

**Case presentation:**

A Scottish school group consisting of 19 individuals visited Uganda during July 2016 with one positive symptomatic case identified on return to the UK. As three of the individuals were not Scottish residents, their data were excluded from this report. Freshwater exposure was noted from taking part in activities which included swimming in the Nile. The Scottish Parasite Diagnostic and Reference Laboratory performed serology testing using sera from 16 Scottish residents to detect IgG towards *Schistosoma* egg antigens. Thirteen were positive despite only one case being symptomatic.

**Conclusion:**

The high positivity rate raised several issues. These included the lack of a robust risk assessment by the travel company organizing the trip, the lack of awareness of schistosomiasis by some individuals, the lack of appropriate and accurate pre-travel advice, and the asymptomatic nature of the infection. This report provides supportive evidence to strengthen the need for improvements to prevent largely asymptomatic cases being missed in future.

## Introduction

*Schistosoma* species are trematode parasites responsible for causing schistosomiasis, identified as a neglected tropical disease by the World Health Organisation [1]. Also known as bilharzia, there are around 230 million people infected worldwide [2, 3]. There are five main species that infect humans, namely *Schistosoma mansoni*, *S. haematobium*, *S. japonicum*, *S. intercalatum* and *S. mekongi*. The life cycle involves the release of free-swimming cercariae from infected snails in freshwater which penetrate human skin. This is most likely to occur during water-based activities, including swimming, water sports and bathing. Endemic areas include Africa, South East Asia, South America and the Middle East [3].

In the UK, tourism to Uganda has increased five-fold in a decade, with 11 % of visitors to the country travelling from the UK in 2013 [4]. Travel to endemic areas is becoming increasingly accessible, and is likely to rise as Scotland continues to have strong links with East Africa. Notably, partnerships through volunteer schemes with Uganda and the Scotland–Malawi partnership promote the development and maintenance of connections between schools, community groups and individuals.

In Scotland, schistosomiasis is the most frequently reported parasitic disease in travellers with over 8000 cases occurring during 2001–2015 and a 25 % positivity rate [5, 6]. Serology testing is advised in those who have been to an endemic region and who have had freshwater exposure. A blood sample is requested at least 8 weeks following the last exposure date, or sooner should symptoms occur. Infection is largely asymptomatic in Scottish travellers, and therefore the number of positive cases in Scotland is likely to be underestimated [5, 6]. Although many travellers are asymptomatic, initially, patients may experience a rash or skin irritation known as swimmers' itch. This is caused by penetration of the skin by the cercariae. Katayama syndrome may occur after 1–2 months with symptoms including fever, chills, cough, urticaria, arthralgias, lymphadenopathy, splenomegaly and abdominal pain. If left untreated, schistosomiasis can lead to potentially life-threatening conditions including advanced hepatic fibrosis disease, portal hypertension, squamous-cell carcinoma of the bladder and central nervous system lesions [7]. In addition, host response to eggs in the genital tract can result in genital lesions and have the potential to cause infertility or adverse pregnancy outcomes. Administration of the drug praziquantel, which kills adult worms, to halt egg production is essential to alleviate symptoms and to prevent long-term disease. Treatment is dependent on a positive serology finding. However, if travellers are not aware of the disease, and are infected but asymptomatic, they will not receive testing and therefore will go untreated.

This report describes the important outcomes of laboratory testing that detected *Schistosoma* antibodies in a Scottish school group. The group comprised 17 students and two teachers who had visited a known endemic region, Uganda, during July 2016. Three of the group resided outside Scotland and are not included in this report.

## Case report

### Investigations

Initially, one patient with gastrointestinal symptoms presented to their general practitioner (GP) and was tested by the Scottish Parasite Diagnostic and Reference Laboratory (SPDRL) for *Schistosoma* antibodies 3 months after returning to the UK. Testing was requested as the individual had been to an endemic region and had experienced freshwater contact. The serum sample from this symptomatic traveller was found to be positive for *Schistosoma* IgG. A further five positive cases were identified 7 months after returning home. All five cases were asymptomatic and had attended their local GP to request testing on their own initiative. All noted recent travel to Uganda and contact with freshwater. Subsequently, the local Health Protection Team (HPT) was notified. The number of cases and the age of the patients suggested a school trip. Through contact with the local education department, the HPT identified a school which had visited Uganda in July 2016, comprising 19 individuals in total. Three were resident outside Scottland and were contacted by the local HPT to recommend testing in their local area. This left 16 members of the group, inclusive of the five cases initially identified.

### Diagnosis

All of the 16 Scottish participants submitted 5–10 ml clotted blood for *Schistosoma* IgG testing by the SPDRL after the initial cluster of five positive cases were identified.

Briefly, 100 µl of patient sample was diluted in wash buffer and added to the well of a 96-well plate coated with *Schistosoma* egg antigens (DRG Diagnostics). Samples were incubated for 1 h at 37 °C before being washed three times in wash buffer. One hundred microlitres of horseradish peroxidase-labelled Protein A conjugate was added to each well and incubated at room temperature for 30 min in the dark. Wells were washed a further three times in wash buffer before the addition of 100 µl tetramethylbenzidine substrate to each well. After incubating for 15 min in the dark at room temperature, 100 µl of stop solution was added to each well before the optical density of each well was read on an ELISA microwell plate reader at a wavelength of 450 nm (Flow Laboratories). The sensitivity and specificity of the assay are given by the manufacturer as 87 and 95 %, respectively. Following detection of *Schistosoma* IgG, faeces and urine samples were submitted for examination of ova using light microscopy wet preparations.

As the majority of Scottish travellers infected with *Schistosoma* species are likely to be asymptomatic, it was advised that all members of the group needed to be tested regardless of the lack of symptoms to ensure appropriate drug therapy was administered to those who tested positive. After notifying individuals to attend their local GPs to have blood samples taken, a total of 13 positive cases were identified (Table 1). Twelve of the 13 positive cases submitted stool and urine samples, which were negative for *Schistosoma* ova. All involved were identified within 1 week of the initial notification, and before complications arose. Information provided by the SPDRL was disseminated to group members through a contact with the school.

**Table 1.** Summary of schistosomiasis cases including water exposure and laboratory findings

Total number on the trip	19 (2 staff and 17 pupils)
Total number of Scottish residents	16 (5 males; 11 females)
Total number of individuals tested by ELISA	16
Total number of ELISA-positive individuals who had swum in the river	13 (5 males; 8 females)
Total number of ELISA-negative individuals	3 (all female; one had swum)

### Treatment

Infectious diseases specialists were contacted to assess and treat the individuals. Due to parent concern and the high positivity rate in this group, a one-time additional clinic for positive patients was arranged. Praziquantel treatment was offered to and accepted by all positive cases. The drug was given on a weight-based dosing schedule. Each patient’s weight was therefore collected by the HPT through the school via email prior to the clinic to ensure that a sufficient amount of this drug was available.

## Discussion

This report highlights several important findings. Firstly, the group was not aware of the risk posed by freshwater swimming. Secondly, they were not aware that the Scottish diagnostic criteria was to test those who have had water exposure and travel to an endemic region. Thirdly, only one patient was symptomatic, which led to the outbreak being identified, despite a large percentage of the group being infected (81 %, 13/16). Two of the three negative patients had not taken part in the freshwater swimming, which was thought to be the source of infection.

Testing had been encouraged through the school before HPT involvement, due to the first positive symptomatic case. The HPT contacted the group through the lead teacher who provided names and contact details, allowing the HPT to remind those who had not been tested to book an appointment with their GP. The lead teacher provided additional information on schistosomiasis to the group from various sources including: (a) NHS Choices website (http://www.nhs.uk/pages/home.aspx), (b) Fit-for-Travel website (http://www.fitfortravel.nhs.uk/home.aspx), (c) email and (d) a private Facebook group.

The purpose of further investigation by the HPT was to encourage testing in the affected school group in order to arrange treatment to prevent more serious long-term complications occurring in the future. This was achieved through communications with the group leader who supplied contact information for those in the group, leading to the identification of further cases. The lead teacher also passed on information provided by the HPT on schistosomiasis to group members.

Schistosomiasis has been identified in at least 74 countries, and has also been identified in those who have travelled to regions not deemed to be endemic. One example was the report describing *S. haematobium*/*S. bovis* hybrid in travellers returning from Corsica, France, in 2014 [8]. In another example, 23, 15 and 10 % of Scottish travellers returning from Europe, North America, and Australia and New Zealand, respectively, between 2001 and 2015 were *Schistosoma-*positive [5]. Links between Scotland and Africa are particularly evident, and where country-specific data were available, 40 % of travellers to Uganda tested positive for schistosomiasis upon their return [5]. Similarly, travel to Uganda accounted for 24.9 % of new cases of schistosomiasis identified in Scotland in 2005–2009 [9].

The data within this investigation mirror a report of a previous outbreak in a Scottish school group, which described a group of travellers to Malawi [10]. In this group, 13/21 (62 %) were found to be positive for schistosomiasis, and only two patients were symptomatic. In the 6 years since this report was published, schistosomiasis continues to be documented in Scottish school groups returning from abroad and has recently been reporting in a school group from Denmark [5, 11]. Advice to schools was first published in 2015 and is available online via the Health Protection Scotland, TRAVAX and Fit-for-Travel websites [12]. In addition, after the outbreak in this report, the Scottish Government wrote to schools via the local education authorities to reinforce previous guidance, highlight the risks posed by schistosomiasis and provide links for further advice. The letter also highlighted that an individual travel health risk assessment must be carried out by a qualified health professional for each person travelling.

This report adds further evidence to support the need to raise awareness of schistosomiasis in returning travellers. In June 2016, the Scottish Schistosomiasis National Advice, Investigation and Liaison (SNAIL) group was launched in response to these outbreaks, and other cases across Scotland. The aims of the SNAIL group include raising awareness of schistosomiasis, particularly amongst higher risk groups [6].

This investigation identified that a robust risk assessment was lacking pre-travel. The school group did not appear to receive information about schistosomiasis from any source prior to travel, including exposure risk in freshwater, and the recommendation for testing after return to the UK. Information has now been disseminated by the Scottish Government for the attention of schools intending travel to at-risk countries. However, while this method will be of use to school groups, it will not capture university groups or volunteer organizations undertaking similar trips. Similarly, around 90 % of travellers to Uganda visit in groups of fewer than four [4] and care should be taken to include smaller groups or lone travellers when developing guidance.

One possible method of targeting travellers is via GPs or private travel clinics used by individuals prior to travelling to receive immunizations/malaria prophylaxis. These would be useful platforms to provide information to those at risk. Although infection risk by country is available on the TRAVAX and Fit-for-Travel websites, the group described in this report remained unaware of the risk until the first member of the group became ill. An analysis of European data found 486/1465 (33 %) cases of imported schistosomiasis from 1997 to 2010 in travellers to endemic areas. Of these travellers, only 18 % received pre-travel advice [13]. This finding is supported by a study which analysed data from 333 confirmed and probable cases of schistosomiasis in Europe from 1999 to 2001 and found that of 147 patients who provided additional data, only 40 % (*n*=59) received pre-travel advice on schistosomiasis [14]. In one Scottish Health Board, out of 77 patients presenting to an Infectious and Communicable Diseases Centre during 2011–2013 with travel-related disease, 21 (27.3 %) had not received a pre-travel consultation [15].

In the outbreak reported here, the only reason cases were identified was due to the one symptomatic case. Enquiring about fellow travellers at the time of diagnosis or treatment could potentially capture other positive cases. The SNAIL group has established enhanced surveillance of schistosomiasis in Scotland since May 2016 which will permit much more in-depth information on every case to be gathered. This will greatly help to make evidence-based improvements to pre- and post-travel advice.

The limitation of this study is that it represents an individual school on a single trip to one country. Therefore, the results described in this report do not necessarily reflect what happens to other school groups heading to endemic regions as the information provided prior to travelling varies depending on, for example, travel company, pre-travel advice offered and previous experiences by individual schools. Nevertheless, this study highlights that outbreaks are still occurring and that despite comprehensive pre-travel advice being readily available online it is not always being provided to the pupils or their parents, which is likely to result in affected individuals failing to receive treatment.

Testing on return is advised at least 8 weeks after the last exposure to freshwater, or sooner if symptoms occur. This allows the adult worms time to mature, to mate and the female to lay eggs. The test detects IgG towards egg antigens and cannot differentiate between a current or previous infection. Although *Schistosoma* antibodies can last for several years after infection [16], it can be assumed that in travelling school groups with limited past exposures, for example only one visit to an endemic region, a positive serology finding is likely to represent a recent infection and hence treatment is advised. Further studies are on-going at the SPDRL to explore alternative testing options that can be used to determine whether infections are current and can be used to determine treatment success.

Serology testing cannot be used to distinguish between species. To do this, microscopy detection of ova in stools and urine is used to examine the distinct differences in egg morphology. In this outbreak, 12/13 positive individuals submitted samples for microscopy, and all were negative. This is supported by data from another recent report of schistosomiasis in a Danish school group where 13 serology-positive cases had no ova identified from their urine and stool samples [11]. Microscopy to detect *Schistosoma* ova lacks sensitivity, and parasite eggs may become trapped in the lipid layer during the concentration process and be discarded. In addition, returning travellers are likely to have a low parasite burden due to limited freshwater exposure, reducing the chances of finding ova in faeces or urine. This results in cases often being serology-positive but microscopy-negative. Treatment is recommended for all serology-positive cases, regardless of their microscopy result.

Variations between Scottish NHS boards exist regarding the timing of sampling, follow-up testing and treatment protocols. The SNAIL group aims to address these areas to improve future patient management. To prevent similar situations from occurring in future, it is crucial to raise awareness of schistosomiasis. It is essential to inform all travellers, not only school groups, of the risks involved before travel and that testing is encouraged on return should they be exposed to freshwater in endemic regions.
